# Evidence for a Critical Role of Catecholamines for Cardiomyocyte Lineage Commitment in Murine Embryonic Stem Cells

**DOI:** 10.1371/journal.pone.0070913

**Published:** 2013-08-02

**Authors:** Martin Lehmann, Filomain Nguemo, Vilas Wagh, Kurt Pfannkuche, Jürgen Hescheler, Michael Reppel

**Affiliations:** 1 Institute for Neurophysiology, University of Cologne, Cologne, Germany; 2 Department of Pediatrics, University of California San Francisco, San Francisco, California, United States of America; 3 Department of Cardiology, Medical University of Lübeck, Lübeck, Germany; Tokai University, Japan

## Abstract

Catecholamine release is known to modulate cardiac output by increasing heart rate. Although much is known about catecholamine function and regulation in adults, little is known about the presence and role of catecholamines during heart development. The present study aimed therefore to evaluate the effects of different catecholamines on early heart development in an *in vitro* setting using embryonic stem (ES) cell-derived cardiomyocytes. Effects of catecholamine depletion induced by reserpine were examined in murine ES cells (line D3, αPIG44) during differentiation. Cardiac differentiation was assessed by immunocytochemistry, qRT-PCR, quantification of beating clusters, flow cytometry and pharmacological approaches. Proliferation was analyzed by EB cross-section measurements, while functionality of cardiomyocytes was studied by extracellular field potential (FP) measurements using microelectrode arrays (MEAs). To further differentiate between substance-specific effects of reserpine and catecholamine action via α- and β-receptors we proved the involvement of adrenergic receptors by application of unspecific α- and β-receptor antagonists. Reserpine treatment led to remarkable down-regulation of cardiac-specific genes, proteins and mesodermal marker genes. In more detail, the average ratio of ∼40% spontaneously beating control clusters was significantly reduced by 100%, 91.1% and 20.0% on days 10, 12, and 14, respectively. Flow cytometry revealed a significant reduction (by 71.6%, n = 11) of eGFP positive CMs after reserpine treatment. By contrast, reserpine did not reduce EB growth while number of neuronal cells in reserpine-treated EBs was significantly increased. MEA measurements of reserpine-treated EBs showed lower FP frequencies and weak responsiveness to adrenergic and muscarinic stimulation. Interestingly we found that developmental inhibition after α- and β-adrenergic blocker application mimicked developmental changes with reserpine. Using several methodological approaches our data suggest that reserpine inhibits cardiac differentiation. Thus catecholamines play a critical role during development.

## Introduction

**Figure 1:**
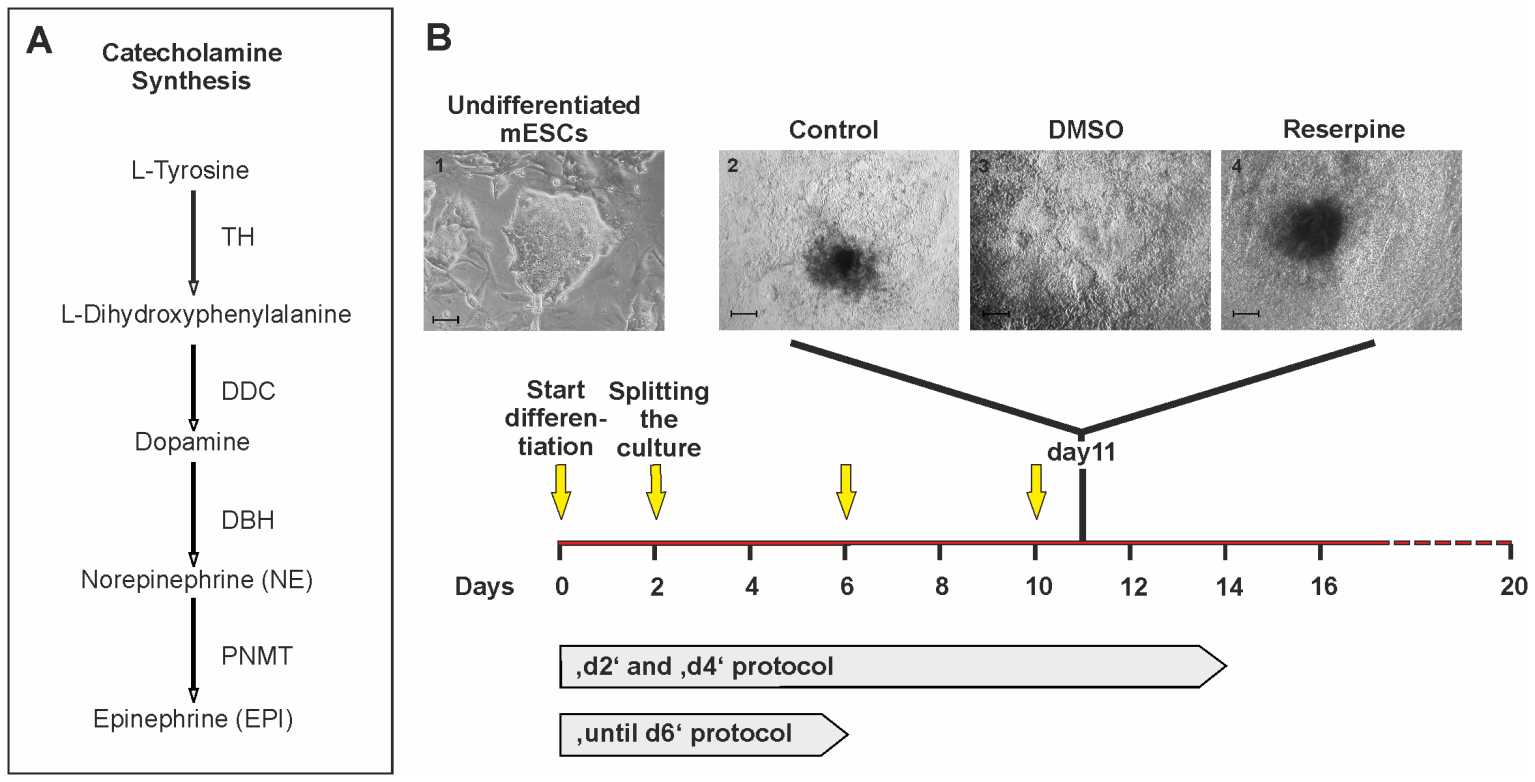
Time course of cell culture and drug application. (A) Schematic overview of catecholamine synthesis and corresponding enzymes (adopted from [3]). (B) Scheme of culture protocols: Yellow arrows indicate the days of medium changes (20% IMDM) and addition of DMSO (1∶1000) or reserpine (10 µM in 1∶1000 DMSO) as used for the ‘d4’ Protocol. (1) Colony of undifferentiated pluripotent D3 αPIG44 murine ESCs on MEFs preceding the start of differentiation (scale bar: 50 µm). (2–4) Control (untreated), solvent control (DMSO; 1∶1000) and reserpine-treated (10 µM in 1∶1000 DMSO) day 11 EBs plated on 0.1% gelatine-coated culture dishes (scale bars 200 µm).

Catecholamines such as dopamine (DA), norepinephrine (NE) and epinephrine (EPI) are important neurotransmitters and hormones with a variety of essential functions in the body. Beside other functions, catecholamines regulate cardiac action by local neuroendocrine secretion [1], [2]. Catecholamine synthesis starts with tyrosine as a precursor which is converted into L-dihydroxyphenylalanine (L-DOPA), DA, norepinephrine NE and EPI by the sequential action of the enzymes tyrosine hydroxylase (TH), DOPA decarboxylase (DDC/AADC), dopamine-β-hydroxylase (DBH) and phenylethanolamine N-methyltransferase (PNMT) (Fig. 1A) [3].

Already in the 1930’s it has been hypothesized that catecholamines might be important regulators of the heart rate even prior to the inception of nerval control [1], [2]. In embryonic chick hearts, catecholamines (NE and EPI) were detected as early as day 3 and L-DOPA and DA were already present from day 1 on [4]. Intrinsic cardiac adrenergic (ICA) cells were later identified in rat [5], mouse [6] and human [7] heart tissue and primary cardiomyocyte isolates. These catecholamine synthesizing ICA cells are present long before neuronal β-adrenergic innervation occurs and were described to have important functions in embryonic heart development and neonatal cardiac β-adrenergic functionality [8]. Moreover, it has been shown that catecholamines are involved in embryonic development *in vivo*. Genetic knockout of TH and DBH, two of the rate-limiting enzymes of catecholamine synthesis, in murine *in vivo* models significantly increased fetal lethality or death shortly after birth [9]–[11]. Although cardiovascular failure was suggested to be a major underlying reason of increased lethality, a detailed insight into neuronal and cardiac interaction of catecholamines during the heart development process is still lacking.

Embryonic stem (ES) cells are pluripotent cells in which differentiation towards certain terminally differentiated lineages can be studied *in vitro*. During differentiation, the cells form spherical aggregates called embryoid bodies (EBs), containing cells of all three germ-layers [12]. For cardiac differentiation the well-characterized D3-derived αPIG44 murine ES cell line is of great value as it expresses enhanced green fluorescent protein (eGFP) and a puromycin resistance gene under control of the cardiac specific alpha myosin heavy chain (αMHC) promoter [13].

Reserpine modulates catecholamine levels in the brain [14]–[17]. It inhibits vesicular transport of catecholamines by the vesicular monoamine transporter (VMAT) leading to a depletion of catecholamine stores. At the same time it prevents re-uptake of catecholamines [18]–[20]. Since extracellular (secreted) or intracellular (leaking from vesicles) catecholamines are quickly degraded by the action of monoamine oxidase (MAO) and catechol-O-methyltransferase (COMT), reserpine completely depletes vesicular packaging and subsequently secretion of the catecholamines dopamine, norepinephrine and epinephrine leaving no extracellular catecholamines for paracrine action (reviewed in [21]). So far, catecholamines in early pre-innervation stage embryos are thought to originate from intrinsic cardiac adrenergic (ICA) cells [5]–[7]. Yet, it has not been investigated whether and how catecholamines modulate differentiation of ES cells toward cardiomyocytes. We therefore hypothesized that catecholamines are already present during the earliest stages of ES cell development and cell fate specification and may efficiently contribute to the differentiation program and function of cardiac cells.

Using a well-established *in vitro* model system of murine ES cells, we provide evidence for an effect of catecholamines very early during differentiation and involvement of α- and β-adrenergic receptors in the differentiation of mES cell-derived cardiomyocytes (ESCMs).

## Materials and Methods

### Cell Culture

The murine D3 αPIG 44 ES cell line was used [22] and passaged as described before [13]. For differentiation we followed a mass culture protocol [23]. In brief, 10^6^ enzymatically dissociated cells were cultivated on a horizontal shaker in non-adhesive dishes in 20%-IMDM (Gibco®, Invitrogen, Karlsruhe, Germany) supplemented with: FCS (20%; v/v), β-Mercaptoethanol (200 µM), non-essential amino acids (1x) for the control group, and dimethylsulfoxide (DMSO; 1∶1000 v/v) or reserpine (10 µM in DMSO) for the solvent control and treated groups, respectively. At day 2 EBs were distributed to fresh 10 cm bacteriological dishes at a density of 1000 EBs/plate in 12 ml culture medium. Afterwards, two differentiation protocols were used (Fig. 1B): 1.) Addition of reserpine every second day with medium change every 6 days (‘d2’ protocol); 2.) Addition of reserpine every fourth day including medium changes at these days (‘d4’ protocol). Since ‘d2’ and ‘d4’ protocols lead to comparable effects of delayed start of beating during ES cell differentiation, we decided to follow the ‘d4’ protocol for further experiments. Control groups with DMSO (solvent control) instead of reserpine were included. In order to estimate the fraction of beating clusters, the EBs were plated on 6-well plates (15–25 EBs per well) at day 6. For experiments elucidating the developmental effects of the α- and β-blockers we used phentolamine (10 µM) and propranolol (5 µM), respectively (both from Sigma Aldrich, Steinheim, Germany). Drugs were refreshed every 24 hrs until day 6, while medium was changed following the ‘d4’ protocol.

### Flow Cytometry

Quantification of eGFP expressing ESCMs was performed by flow cytometry. 100–200 EBs were harvested at corresponding days, washed in Ca^2+^- and Mg^2+^-free phosphate-buffered saline (PBS −/−, Invitrogen, Karlsruhe, Germany) and enzymatically dissociated to a single-cell suspension using 2 ml trypsin/EDTA for 10 min. Trypsin was inhibited by addition of 10 ml 20%-IMDM and the cell suspension was filtered by passing through a 40 µm cell strainer. For analysis cells were washed in PBS and subsequently resuspended in 1 ml cell-wash solution (BD Biosciences, Franklin Lakes, NJ USA). Per measurement 50,000 cells were sampled. Data of eGFP Expression in ESCMs were acquired using a FACScan flow-cytometer (BD Biosciences, Franklin Lakes, NJ USA). Dead cells were stained with propidium iodide (PI). Data analysis was performed using Cyflogic (v 1.2.1, Turku, Finland).

### Immunohistochemistry

Two days prior to staining EBs were plated on 1% gelatine-coated cover slips and incubated at 37°C in 20% IMDM for control groups or supplemented with 1∶1000 DMSO (v/v) or 10 µM reserpine for treated groups, respectively. Finally, EBs were fixated with 4% paraformaldehyde for 30 min at room temperature. Preparations were permeabilized using permeabilisation solution (0.25%TritonX, 0.5 M NH_4_Cl). After washing with PBS, the preparations were treated with 1∶10 Roti®Block (Roth, Karlsruhe, Germany) to prevent cross binding of antibodies. Following primary antibodies diluted in 1∶100 Roti®Block (Roth) solution were used as (1) cardiac markers: anti-αActinin (clone EA53, 1∶800; Sigma-Aldrich; Prod# A7811), anti-cardiac Troponin T (1∶1000; Thermo Scientific; Prod# MS-295-P1), (2) neural markers: anti-β-III-Tubulin (1∶1000; GeneTex; Prod# GTX27751), (3) Catecholamine synthesis: anti-dopamine-β-hydroxylase (1∶200; GeneTex; Prod# 19353). Incubation with primary antibodies was performed overnight at 4°C. After six washing steps, samples were incubated with secondary antibody for 2 hrs at 4°C. As secondary antibodies we used: goat-anti-mouse Alexa Fluor 555 (1∶1000; Invitrogen; Prod# A21081), donkey-anti-sheep Alexa Fluor 647 (1∶200; Molecular Probes). Nuclei were co-stained with nucleic acid stain Hoechst33342 (1 µg/ml; Sigma Aldrich; Prod# B2261). Stained samples were embedded in Prolong Gold (Invitrogen, Karlsruhe, Germany) and imaged on a Zeiss Axiovert 200 (Carl Zeiss, Jena, Germany). For analysis the Carl Zeiss software AxioVision LE 4.5 was used.

### RNA Isolation

For total RNA isolation EBs were harvested from puromycin and non-puromycin treated mass cultures at days 1–4, 6, 8, 10 and 14 of differentiation. Together with samples of undifferentiated αPIG44 ES cells and mouse embryonic fibroblasts (MEF) all samples were collected in TriZol® Reagent (Invitrogen, Karlsruhe, Germany) and frozen. According to manufacturer’s guidelines RNA was isolated and stored at −20°C until further use. Prior to cDNA synthesis potentially contaminating genomic DNA was digested using DNase I (1 U/µl; Amplification Grade, Invitrogen, Karlsruhe, Germany).

### cDNA Synthesis

cDNA was synthesized from 2 µg of RNA using the SuperScript**®**Vilo™ cDNA Synthesis Kit (Invitrogen, Karlsruhe, Germany) according to the manufacturer’s guidelines. Briefly, reverse transcription was performed at 42°C for 60 min and the final inactivation step both being performed in a peqSTAR 96 Universal Thermocycler (PEQLAB Biotechnologie GmbH, Erlangen, Germany).

### Quantitative-RT-PCR (qRT-PCR)

Real Time PCR analysis was carried out on ABI-7500 Fast PCR system (Applied Biosystems, Weiterstadt, Germany). The PCR reaction consisted of 12.5 µl SYBR Green PCR master mix (QuantiFAST, Qiagen, Hilden, Germany), 1 µl of primer pair (0.2 µM each) and 1 µl of cDNA template made to a final volume of 25 µl. The standard conditions for PCR were used, 95°C/5 min Taq activation, 40 cycles of 95°C/10 sec. and 60°C/30sec. A melting curve was produced to verify single PCR product amplification. The mRNA levels were normalized against β-actin levels and calculated using a relative quantitation with the cycle threshold method (ΔΔCt method) by 7500 Fast System SDS software 1.4.0. (Applied Biosystems, Carlsbad, Ca, USA). The primer sequences used in the PCR analysis are shown in Table 1. qRT-PCR reactions were carried out in technical triplicates for each sample. Negative controls with H_2_O as template were included. Results are shown as mean ± SD. Statistical analysis was done using paired Student’s *t*-test. P-values of ≤0.05 (*) were considered significant.

**Table 1:** Primers for qRT-PCR and sqRT-PCR (Fig. 3A–F).

Gene	F-primer	R-primer
*Zpf42(Rex1)*	GAGACTGAGGAAGATGGCTTCC	CTGGCGAGAAAGGTTTTGCTCC
*Oct4*	CAGCAGATCACTCACATCGCCA	GCCTCATACTCTTCTCGTTGGG
*Nanog*	GAACGCCTCATCAATGCCTGCA	GAATCAGGGCTGCCTTGAAGAG
*Fgf5*	AGAGTGGGCATCGGTTTCCATC	CCTACAATCCCCTGAGACACAG
*Map2*	GCTGTAGCAGTCCTGAAAGGTG	CTTCCTCCACTGTGGCTGTTTG
*Tubb3*	CATCAGCGATGAGCACGGCATA	GGTTCCAAGTCCACCAGAATGG
*Nes*	AGGAGAAGCAGGGTCTACAGAG	AGTTCTCAGCCTCCAGCAGAGT
*Sox17*	GCCGATGAACGCCTTTATGGTG	TCTCTGCCAAGGTCAACGCCTT
*Fox2a*	TGGAGCTGTGTATGGTCCTGAG	GTTGGGTAAGGGAAGCCAGGAA
*Hnf4*	TGCGAACTCCTTCTGGATGACC	CAGCACGTCCTTAAACACCATGG
*Alb*	CAGTGTTGTGCAGAGGCTGACA	GGAGCACTTCATTCTCTGACGG
*Afp*	GCTCACATCCACGAGGAGTGTT	CAGAAGCCTAGTTGGATCATGGG
*Th*	TGCACACAGTACATCCGTCATGC	GCAAATGTGCGGTCAGCCAACA
*Ddc*	GGAGCCAGAAACATACGAGGAC	GCATGTCTGCAAGCATAGCTGG
*Dbh*	GAGACTGCCTTTGTGTTGACCG	CGAGCACAGTAACCACCTTCCT
*Pnmt1*	CAGGAGCCTTTGACTGGAGTGT	TCGATAGGCAGGACTCGCTTCA
*Myh6*	GCTGGAAGATGAGTGCTCAGAG	CCAGCCATCTCCTCTGTTAGGT
*Mlc2a*	AGGAAGCCATCCTGAGTGCCTT	CATGGGTGTCAGCGCAAACAGT
*Nkx2.5*	TGCTCTCCTGCTTTCCCAGCC	CTTTGTCCAGCTCCACTGCCTT
*Gata4*	GCCTCTATCACAAGATGAACGGC	TACAGGCTCACCCTCGGCATTA
*T-bra*	ATCCACCCAGACTCGCCCAATT	CTCTCACGATGTGAATCCGAGG
*ACTB*	CATTGCTGACAGGATGCAGAAGG	TGCTGGAAGGTGGACAGTGAGG
*Adrb1*	GACGCTCACCAACCTCTTCA	ACTTGGGGTCGTTGTAGCAG
*Adrb2*	TGAAGGAAGATTCCACGCCC	CCGTTCTGCCGTTGCTATTG
*Adrb3*	AGGCACAGGAATGCCACTCCAA	GCTTAGCCACAACGAACACTCG
*Adra1a*	GAGTTTGAGGGCTCAGCAGT	AGTGACTCTCAACTTGGCCG
*Adra1b*	AGAACCCTTCTACGCCCTCT	GGAGCACGGGTAGATGATGG
*Adr1d*	ATCGTGGTCATGTACTGCCG	GACGCCCTCTGATGGTTTCA
*Adra2a*	CAGGTGACACTGACGCTGGTTT	GACACCAGGAAGAGGTTTTGGG
*Adra2b*	CCCTCATCTACAAGGGCGAC	TTCGGGATCTTCAGGGGTCT
*Adra2c*	TCACCGTGGTAGGCAATGTG	GCGGTAGAACGAGACGAGAG
*Gapdh*	GGCTCATGACCACAGTCCAT	ACCTTGCCCACAGCCTTG

### Semiquantitative-reverse Transcription PCR (sqRT-PCR)

sqRT_PCR amplification was performed using the DreamTaq™ Green PCR kit (Fermentas, St. Leon-Rot, Germany). Following manufacturer’s guidelines, PCR reactions contained: DreamTaq™ Green PCR Master Mix (2x), 1 µM of forward and reverse primer (see table 1), 2 µl cDNA in a total volume of 25 µl. PCR protocols consisted of an initial denaturation (95°C, 2 min) followed by respective numbers of cycles of denaturation (95°C, 30 sec), annealing (60°C, 30 sec) and extension (72°C, 30 to 45 sec). Final extension was allowed at 72°C for another 10 min (all performed in a peqSTAR 96 Universal Thermocycler, PEQLAB Biotechnologie GmbH, Erlangen, Germany). Finally, samples were run on1.5% agarose gels and pictures were taken for analysis.

### Extracellular Recordings using Microelectrode Arrays (MEA)

Extracellular recordings of field potentials (FP) with MEAs were performed using the Multichannel Systems 1060-Inv-BC amplifier and data acquisition system (Multichannel Systems, Reutlingen, Germany). Substrate-integrated MEA culture dishes contain 60 Titanium Nitride coated gold electrodes (30 µm diameter) arranged in an 8×8 electrode grid with an inter-electrode distance of 200 µm, allowing simultaneous recording of extracellular FPs from all electrodes at a sampling rate of 1 to 50 kHz by the use of the MEA amplifier system. Standard measurements were performed at a sampling rate of 2 kHz in IMDM. For the extracellular recordings we used either beating EBs directly from the mass culture or EBs that were prior plated on 1% gelatine-coated multi-well culture dishes at day 6 of differentiation. Beating clusters were mechanically dissected (if grown on multi-wells), transferred to MEA dishes and subsequently allowed to attach for 2 days in the incubator. MEA culture dishes were coated using a 1∶200 solution of fibronectin (1 mg/ml) and gelatine (0.1%). During measurements temperature was kept constant at 37°C. MEA data were analyzed applying a software tool based on LabView (National Instruments, Austin, Tx USA) that was created by the first author. The tool allows for analysis of MEA signals in order to quantify interspike intervals (ISIs). ISIs were defined as the time interval between two consecutive FP minima (FP_min_) thus providing a read out for beating frequency [24], [25].

### Data Analysis

For data analysis student’s paired or unpaired t-test and one-way ANOVA were used, where applicable. Error bars represent the standard error of the means (s.e.m.) if not stated otherwise. P-values ≤0.05 (*) were considered significant. For statistical analysis GraphPad InStat Version 3.10 and GraphPad Prism® (GraphPad Software, Inc., San Diego, Ca USA, www.graphpad.com) were used.

## Results

### Reserpine Inhibits Cardiac Differentiation

**Figure 2:**
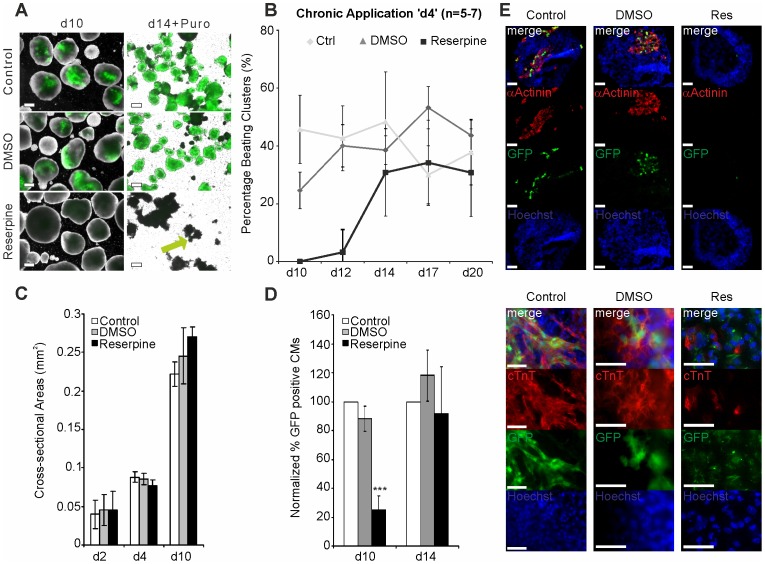
Effect of reserpine on proliferation and cardiomyocyte differentiation. (A) Microscopy images of EBs at day 10 and 14 of differentiation (day 14 EBs were purified with puromycine). eGFP positive CMs containing clusters in control, DMSO (solvent control) and reserpine-treated EBs are shown (green arrow in reserpine-treated). Scale bars: 200 µm. (B) Time course of appearing spontaneously beating clusters within plated EBs at indicated time points. (C) EB proliferation was analyzed based on cross sectional areas (mm^2^) from phase contrast pictures captured at days 2, 4 and 10 after differentiation start (n = 3). (D) Percentage of eGFP positive CMs from control, DMSO and reserpine treated EBs at day 10 (n = 11; *** p<0.01) and day 14 (n = 7) of differentiation derived from flow cytometry. (E) Immunocytochemical staining of EBs at day 11 of differentiation for cardiac marker proteins: eGFP positive cardiomyocytes (green), Hoechst 33342 stained nuclei (blue) and cardiac αActinin (red; top panel) or cardiac TroponinC (red; bottom panel). Top row represents overlay pictures. Scale bars: 50 µm.

D3 αPIG44 ES cells harbor a vector expressing eGFP and a puromycin resistance gene under the control of a murine *αMHC* promoter. When clustered in EBs the cells differentiate within 8 days and form spontaneous contracting cardiomyocytes among other cell types. Fig. 2A shows examples of EBs differentiated under control (untreated and DMSO-treated) and reserpine-treated conditions at day 10 as well as cardiac clusters at day 14 of differentiation that have been treated with puromycin to isolate cardiomyocytes (Suppl. 2: pictures of day 2 and day 4 EBs are shown). We found that reserpine treatment led to a significantly lower number of EBs expressing eGFP as an indicator of differentiated cardiomyocytes at day 10. Simultaneously a reduction of cardiac cluster size within the EBs was observed. Purification of control EBs yielded almost pure normal sized cardiac clusters, whereas after reserpine treatment only very small eGFP positive clusters (green arrow) were found (Fig. 2A, right column).

Additionally, when calculating the ratio of beating to non-beating EBs in cultures following the ‘day 4′ protocol (Fig. 1A), we observed the first appearance of control beating EBs at day 8–9 (data not shown). At day 10, 25±7% spontaneously beating EBs were found in the untreated control group (Fig. 2B). The DMSO control group yielded 46±12% beating EBs (from n = 6 independent differentiations). No beating was detected in reserpine-treated EBs at day 10. In reserpine-treated EBs, beating clusters appeared by day 12 of differentiation with very low incidence (3.2±7.8%, n = 7), whereas in both control groups at least 40% beating EBs were found at day 12. Moreover, the untreated and DMSO-treated groups showed maximal percentages of beating clusters of 35.8±7.3% and 43±12%, respectively, whereas reserpine-treated EBs showed maximum percentage of 34±15% at day 17 of differentiation. After day 20 the rate of spontaneous beating declined similarly in all three groups (data not shown). To exclude reserpine-induced modulation of cell proliferation and cytotoxic effects we analyzed cross section areas of 127–230 EBs from each group of 3–4 independent differentiations at different stages during differentiation (Fig. 2C). We found no significant difference between the groups at day 2 with a mean area of 0.040±0.019 mm^2^ in the untreated, 0.045±0.020 mm^2^ in the DMSO-treated group and 0.046±0.024 mm^2^ after reserpine treatment. Neither untreated (0.088±0.0064 mm^2^), DMSO-treated (0.086±0.0075 mm^2^), nor reserpine-treated EBs (0.077±0.0067 mm^2^) showed significant differences at day 4 of differentiation. Also at day 10 EB size did not differ significantly (0.222±0.0154 mm^2^, 0.246±0.0362 mm^2^, and 0.270±0.0126 mm^2^, respectively, p>0.05). As expected, an exponential growth of EB size could be observed in all three groups during the course of differentiation (Fig. 2B) indicating normal cell proliferation. Moreover, we confirmed the reserpine-induced decrease in eGFP expressing CM numbers in EBs using flow cytometry and quantified it by normalization toward CM numbers in control EBs (Fig. 2D; Suppl. 1). At day 10 no significant differences were found between untreated cells and DMSO-treated EBs (100% *vs.* 87.3±9%, n = 11 independent differentiations, p>0.05). According to the morphological findings (Fig. 2A), we found that in reserpine-treated EBs eGFP expressing CMs were reduced to 28.4±13.8% at day 10 (n = 11, p<0.001). At day 14 of differentiation reserpine-treated EBs contained 8% less eGFP positive CMs than control EBs (92±32% *vs.* 100%, n = 7 differentiations, p>0.05). Also DMSO-treated EBs did not show significant differences as compared to controls (118±18%, n = 7, p>0.05). To study the development of the structural organization of cardiomyocytes during continuous presence of reserpine, we performed immunocytochemical stainings of the cardiac specific proteins αActinin and cardiac TroponinT (cTnT) in EBs at day 11 of differentiation. As shown in Fig. 2E, both, αActinin and cTnT, were co-expressed with eGFP in untreated and DMSO-treated EBs. Higher magnification showed the typical cross striation pattern for both proteins. However, reserpine-treated EBs showed markedly less cells positively stained for αActinin and cTnT or complete absence of those cells within EBs, suggesting that reserpine strongly inhibits expression of cardiac proteins during murine ES cell differentiation.

### Reserpine Affects Gene Expression

**Figure 3:**
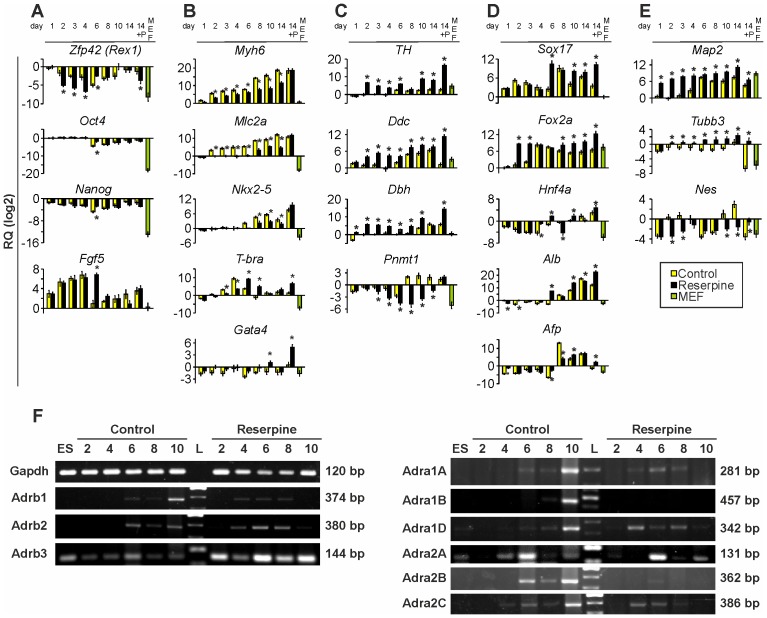
Quantitative real-time PCR analysis during ES cell differentiation. Time course of relative expression of (A) pluripotency genes (*Zfp42, Oct4, Nanog*) and differentiation marker (*Fgf5*), (B) cardiac specific genes (*Myh6*, *Mlc2a*) and mesodermal markers (*Nkx2-5, Gata4* and *T-bra*), (C) catecholamine synthesis genes (*TH, Ddc, Dbh, Pnmt*), (D), endodermal marker genes (*Sox17, Fox2a, Hnf4a, Alb, Afp*), (E) ectodermal and neuronal markers (*Map2, Tubb3, Nes*). 14+P: d14 puromycin purified clusters; MEF: mouse embryonic fibroblasts. Values represent relative quantities (RQ) plotted on a log2 scale. Primers are listed in Table 1. (F) Semiquantitative reverse-transcription PCRs from ES cells (ES), control and reserpine-treated EBs during differentiation from day 2 to day 10 representative of the gene expression of α- and β-AR subtypes (α_1A_, α_1B_, α_1D_, α_2A_, α_2B_, α_2C_, β_1_, β_2_, β_3_; *Gapdh* was used as house-keeping control). L: DNA Ladder. Amplified fragment sizes are stated on the right of each pane.

To further elucidate how reserpine inhibits differentiation of ES cell towards CMs, we used qRT-PCR analysis to investigate the expression of several groups of marker genes. The pluripotency gene *Rex1* was significantly stronger down-regulated in the first 4 days of differentiation (Fig. 3A), suggesting that reserpine-treated cells lose their pluripotency properties earlier than control cells. In contrast, *Oct4* as well as *Nanog* were not significantly affected. *Fgf-5* expression was strongly increased in both groups. Although after day 4 in the control group *Fgf-5* was dramatically down-regulated [26], this down-regulation could only be observed after day 6 in the reserpine group (Fig. 3A). This in turn suggests that reserpine treatment either prolonged the phase in which cells differentiate or that it hampered the differentiation process. As expected, Zfp42 (Rex1), *Oct4, Nanog* and *Fgf-5* expression levels were very low in MEF control samples.

Furthermore, the expression of the cardiac specific genes myosin heavy chain (*Myh6*) and atrial myosin light chain 2 (*Mlc2a*) should elucidate how strong reserpine affected CM differentiation on the gene expression level. qRT-PCR results of the cardiac genes revealed (Fig. 3B) that Myh6 showed a ∼250-fold reduced level of expression in reserpine-treated EBs at day 10. Mlc2a did not even seem to be expressed until day 6–8 and expression levels never reached control EB levels (up to ∼130-fold reduced expression at day 14). These results underline the finding of reserpine-induced inhibited cardiac differentiation. Purified CMs of control and treated groups showed almost equal levels of gene expression. MEFs did not express these cardiac genes. To investigate whether only cardiac genes or also mesodermal or early CM gene expression is altered, we tested the expression of the specific markers homebox protein Nkx2-5, T-brachyury (T-bra) and Gata binding factor 4 (Gata4) (Fig. 3B). Initial transcription of Nkx2-5 was found to be delayed by approximately 2 days (day 8 instead of 6) in reserpine-treated EBs. Peaking at day 10 in both groups, Nkx2-5 expression was significantly lower in reserpine-treated EBs at each time point. Accordingly, initiation of T-bra expression was also delayed by about one day. The maximum expression of T-bra was reached at day 6 in reserpine-treated EBs instead of day 4 in control EBs. In purified day 14 CMs T-bra and Gata4 expression showed higher levels in the reserpine group, whereas Nkx2-5 expression was not altered. Nkx2-5, Gata4 and T-bra were only very weakly expressed in MEFs.

To further understand the importance of catecholamines during cardiac differentiation, the expression of catecholamine synthesis genes during differentiation was studied (Fig. 3C). We found no significant expression of Th and Ddc until day 6 in controls. For Dbh and Pnmt, even later expression was detected (around day 8–10). In contrast, reserpine-treated EBs showed up-regulation of Th, Ddc and Dbh already at day 2. Pnmt showed low expression and decreasing levels during differentiation in reserpine-treated EBs, whereas in controls up-regulation of Pnmt expression started after day 8.

Examining the reserpine effect on endodermal differentiation and to further investigate whether delayed or inhibited mesodermal and cardiac differentiation might be compensated through alternative lineage differentiation, we tested the expression of endodermal marker genes *Sox17, Foxa2,* alpha-fetoprotein (*Afp*), hepatocyte nuclear factor 4a (*Hnf4a*) and albumin (*Alb*) (Fig. 3D). Amongst the tested endodermal genes only the transcription factors *Sox17* and *Foxa2* show altered expression patterns. Most striking is the earlier up-regulation observed for both genes at day 6 instead of day 8 and day 2 instead of day 4, respectively, whereas, the expression of later endoderm-derived liver tissue-inducing genes *Afp*, *Hnf4a* and *Alb* revealed no such effect of reserpine. In MEFs, expression of all endodermal marker genes tested here, except for *Foxa2*, is at a level of pluripotent ES cells or below. These results suggest that reserpine affects certain aspects of early endodermal development.

We further studied the time course of the expression of α- and β-AR subtypes using sq-RT-PCR (Fig. 3F) comparing control and reserpine-treated EBs. We found that in control EBs the β_1_- and the β_2_-AR expression started at day 6 while after reserpine treatment first expression was detected at day 4. However, at day 10 β_1_- and β_2_-ARs were absent in reserpine-treated EBs. Interestingly, the β_3_-ARs were already expressed in ES cells. We also found that β_3_ receptor levels were increased in reserpine-treated EBs. All α_1_-ARs were clearly expressed at day 10 in the control group, whereas no expression of α_1_-ARs was found in reserpine-treated EBs. A distinct transient expression of α_1A_ and α_1D_ was detectable between days 4–8. α_1B_ was not expressed at any time in reserpine-treated EBs. Expression of the α_2A_ receptor was similar in both groups of EBs, while α_2B_ expression strongly differed. In control EBs expression of α_2B_ receptors started by day 6 while in reserpine-treated EBs no expression was detectable. Like the β_1_- and β_2_ receptors, α_2C_ was expressed at day 4 but absent at day 10 in reserpine-treated EBs while in control EBs its expression started at day 6 and increased until day 10.

### Reserpine Induces Neuronal Differentiation

**Figure 4:**
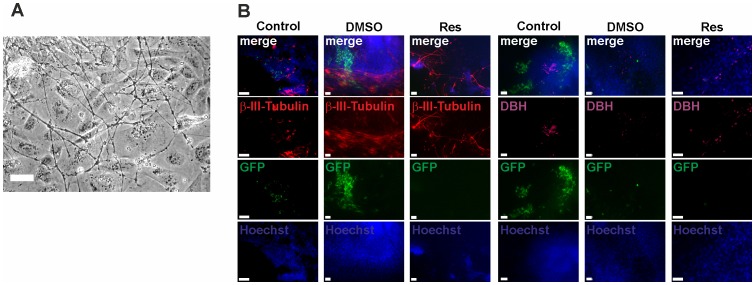
Effect of reserpine on ectodermal and mesodermal marker expression during ES cell differentiation toward CMs. (A) Representative phase contrast image of reserpine-treated EB showing cells with typical neuronal morphology. Scale bar: 50 µm. (B) Immunolocalization of (left) neuronal marker β-III-Tubulin (red) and (right) catecholamine synthesis enzyme dopamine-β-hydroxylase (DBH; violet) in day 11 EBs: eGFP positive cardiomyocytes (green), Hoechst 33342 stained nuclei (blue). Top row represents overlay pictures.

Investigating ectodermal marker gene expression using qRT-PCR (Fig. 3E), we found that in control EBs microtubule-associated protein 2 (*Map2*) expression started around day 4 and was at a constant level between day 6 and 14. In reserpine-treated EBs *Map2* was expressed earlier, i.e. at day 1. β-III-tubulin (*Tubb3*), a valuable neuronal marker [27], was expressed from day 6 on in reserpine-treated cells, whereas in control EBs it appeared to be absent. Nestin (*Nes*), a neural progenitor marker [28], [29], was expressed from day 10 on. Unexpectedly, in reserpine-treated EBs *Nes* expression was rather low and therefore not in accordance with expression patterns of *Map2* and *Tubb3*. Nevertheless, Fig. 4A depicts a representative phase contrast picture of a day 11 reserpine-treated EB showing neuronal cells. In control EBs similar numbers of neuronal cells have never been observed. To confirm the molecular and microscopic findings, immunocytochemical stainings with antibodies specific for TUBB3 and DBH were carried out (Fig. 4B). In all day 11 EBs TUBB3 positive cells were found. Cell morphology obviously showed that control and reserpine-treated cells were different. While the cells in control EBs were of round shape, reserpine-treated cells showed a high number of neuronal-like cells (Fig. 4B, right panel). DBH expressing cells were of round shape [7] and were found in all three groups showing slightly increased numbers in reserpine-treated EBs (Fig. 4B, right panel). Taken together our qRT-PCR and immunocytochemistry experiments investigating the presence of the DBH enzyme showed congruent findings (Fig. 3C and Fig. 4B): 1. qRT-PCR for *Dbh* at day 10 proved elevated expression levels in reserpine-treated EBs confirming IHC results (from day 11 EBs); 2. Reserpine-treated purified day 14 CMs had distinctly increased *Dbh* expression, suggesting that *Dbh* synthesizing cells were present.

### β-adrenergic and Muscarinic Signaling Pathway are Functionally Expressed

**Figure 5:**
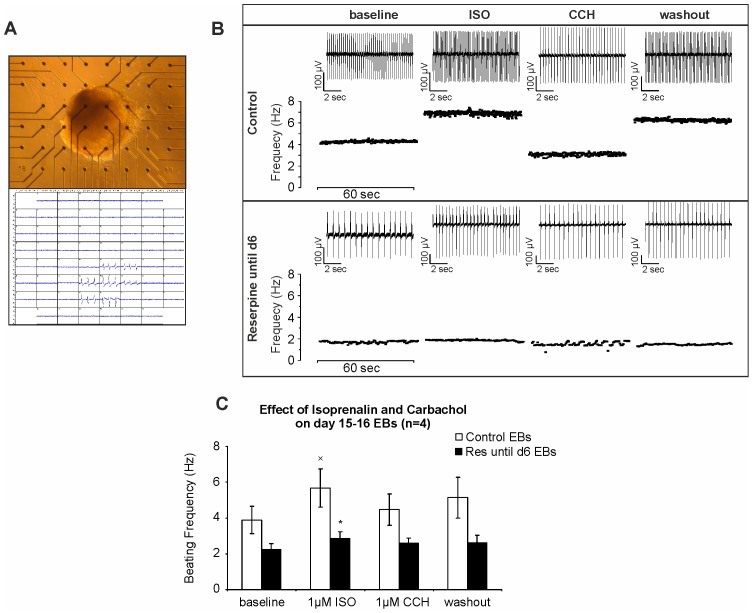
Comparison of β-adrenergic and muscarinic modulation of beating rate in control and reserpine-treated ES cell-derived cardiomyocytes. (A) Spontaneously contracting EB plated on MEA. (B) Representative beating frequencies demonstrating effects of ß-adrenergic agonist isoprenaline (ISO, 1 µM) and muscarinic agonist carbachol (CCH, 1 µM) on cardiac clusters generated under control (top panel) and reserpine-treated (bottom panel) conditions. Original FP traces (10 sec) of each indicated condition are showcased (on top of each plot). (C) Statistical analysis of FP frequencies in MEA measurements (n = 4). (^x^p<0.05: significant difference between baseline and 1 µM ISO in ctrl EBs; *p<0.05: significant difference between ctrl and reserpine-treated EB under ISO (1 µM)).

Since β-adrenergic and muscarinic signaling is crucial for heart function, we investigated whether both systems are functionally expressed and active in spontaneously contracting EBs generated under influence of reserpine. For these experiments, reserpine was applied until day 6 of differentiation before contracting eGFP positive EBs (day 14–16) were plated onto microelectrode arrays (MEAs) (Fig. 5A). The analysis of FPs (Fig. 5C) revealed strong reduction of beating frequencies at baseline conditions in reserpine-treated EBs (2.23±0.34 Hz, n = 4) as compared to control EBs (3.89±0.75 Hz, n = 4). In control EBs, application of ISO (1 µM) led to a significant increase (5.67±1.06 Hz, *p<0.05), whereas CCH (1 µM) decreased beating frequencies (4.47±1.14 Hz, n = 4), suggesting functional expression of both, ß-adrenergic and muscarinic pathways. However, application of the same concentration of ISO and CCH on reserpine-treated EBs did not reveal significant changes in beating frequency (2.87±0.36 Hz for ISO and 2.58±0.41 Hz after CCH application, n = 4). Statistical analysis confirmed the significant positive chronotropic effect of ISO in control EBs as compared to reserpine-treated ones (Fig. 5C, ^x^p<0.05). This finding suggests that reserpine application during differentiation may have changed the availability and/or activity of the β-adrenergic receptors or may have inhibited their expression.

**Figure 6:**
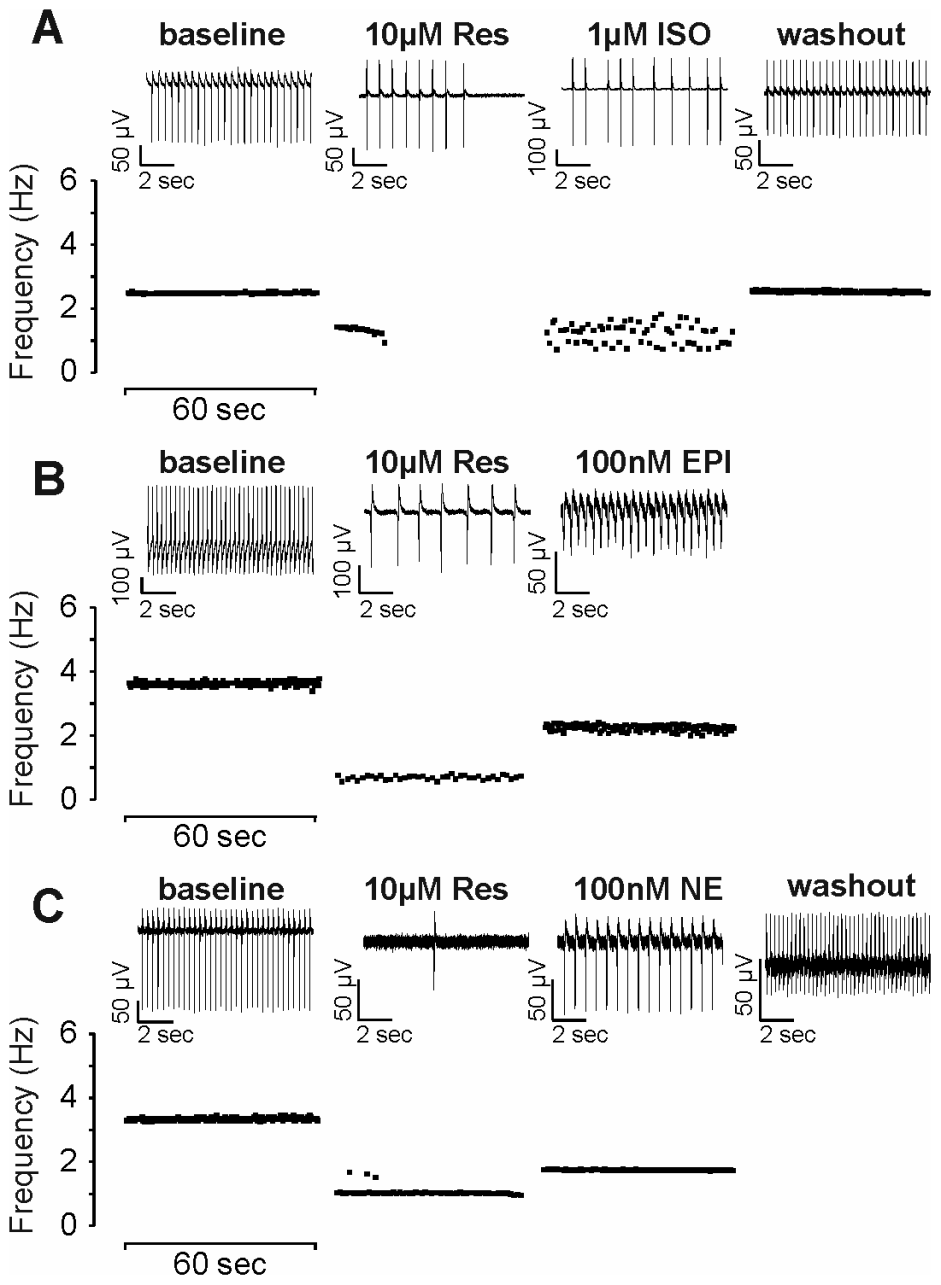
Effect of catecholamines on spontaneously beating clusters of EBs after acute application of reserpine. Representative FP frequencies of day 11 to 12 beating cardiac clusters under acute presence of reserpine (10 µM) and after recovery with adrenergic receptor agonists (A) ISO (1 µM), (B) epinephrine (EPI, 100 nM) and (C) norepinephrine (NE, 100 nM). EBs were incubated with reserpine until maximum negative chronotropic effects were observed (between 8–30 min) and then co-applied with the adrenergic agonist as stated. Depicted are frequency-time plots of representative 60 sec intervals during the experiment. 10 sec FP traces are showcased on top of each. Note: After rescue with epinephrine no washout could be recorded. Full summary of all EBs measured is shown in Table 2.

To determine if the marked changes in beating frequencies of EBs treated acutely with reserpine is associated with inhibition of adrenergic signaling we investigated the effect of the β-adrenergic agonist isoproterenol (ISO) and exogenous catecholamines such as NE and EPI on day 11–12 spontaneously contracting control EBs by MEA. Application of reserpine induced negative chronotropy and arrhythmia (Fig. 6A-C). The most prominent effect was observed 8–30 min after drug application. Subsequent application of ISO (1 µM), EPI (0.1 µM) and NE (1 µM) reversed the chronotropic effect of reserpine. Rescue experiments with ISO resulted in significant recovery of the beating rates in 5 out of 7 EBs. Moreover, addition of EPI and NE also rescued the reserpine effect (in 5 out of 5 and 3 out of 4 EBs, respectively, Table 2).

**Table 2:** Summary of all acute reserpine application experiments and adrenergic receptor (AR) agonist stimulated recovery (from Fig. 6A–C).

		Reserpine				AR Agonist			Washout				
	N =	Negative	Stop	No effect	Arrhythmia	Yes	No	Undefined	Yes	No		Undefined	
		chronotropy											
Res+ISO	7	4	3	0	3	5	1	1	5		2		0
Res+EPI	5	3	2	0	2	5	0	0	2		3		0
Res+NE	4	2	2	0	0	3	1	0	2		2		0
Overview	16	9	7	0	5	13	2	1	12		7		0

Shown are the numbers of control EBs and their responses during: 1. Reserpine: Acute Reserpine application (e.g. EBs responding by negative chronotropy, stop of beating, etc.). 2. AR Agonist: AR Agonist stimulation following reserpine application (EBs which reacted with positive (yes), neutral (no) or very weak (undefined) chronotropy). 3. Washout: After repeated medium changes (showing recovery toward baseline beating rate). Altogether 16 EBs were tested and their responses are further represented as overview (bottom row).

### α- and β-AR Antagonists Mimic the Reserpine Effect

**Figure 7:**
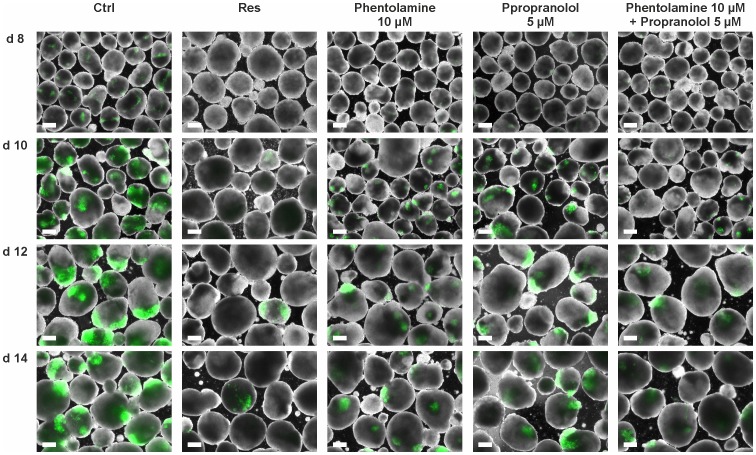
Blocking of α- and β-ARs mimics the reserpine-induced effect on cardiomyogenesis. Fluorescence pictures of EBs treated with the unspecific α- and β-blockers phentolamine (10 µM) and propranolol (5 µM) and the combination of both showing the respective expression of GFP positive CMs on days 8 to 14 of differentiation. Left and the second to left column representing control and reserpine-treated EBs differentiated from the same passage, respectively. (Scale bars: 200 µm).

The negative effect on cardiomyogenesis is hypothetically caused by the catecholamine depleting effect of reserpine and a subsequent lack of α- or β-adrenergic signaling. We therefore investigated whether blocking of α- and β-ARs would also suppress cardiac development in our model. Applying the α-AR antagonist phentolamine and the β-AR antagonist propranolol as well as applying the combination of both we found similar results as after reserpine treatment (Fig. 7). Phentolamine (10 µM; Fig. 7, 3rd column) as well as propranolol (5 µM; Fig. 7, 4th column) reduced the number of GFP expressing CMs in treated EBs compared to control EBs (Fig. 7, 1st column) between day 8–14 of differentiation. α-AR inhibition seemed to exhibit a more negative cardiogenic effect than β-AR inhibition. Combined application of phentolamine and propranolol (Fig. 7, right column) further reduced of the number of CMs, resembling the reserpine effect (Fig. 7, 2nd column). Especially the time-point of cardiac cluster appearance was altered. In control experiments, as well as in the phentolamine- and the propranolol-treatment groups, CMs were found to be clustered while in the α- plus β-AR inhibition group CMs were not only significantly reduced but also less organized at day 10. EB size and shape of all groups was comparable suggesting normal growth and proliferation.

## Discussion

Catecholamines are known to be essential for regulation of mammalian heart function and, in combination with other neuronal and hormonal systems, for several other physiological processes. Moreover, synthesized in different cell types and acting as neurotransmitters as well as hormones, catecholamines have further been shown to be crucial for embryonic development [9]–[11], [30]. However, despite of the significant research efforts to investigate the role of catecholamines in the adult system, their specific role and significance during embryonic cardiomyogenesis remains poorly understood.

We here demonstrate that reserpine remarkably inhibited the cardiac lineage and subsequently delayed differentiation of mouse ES cells towards cardiomyocytes. This delay and inhibition can be attributed to deficiency in the transcriptional regulation and expression of specific germ layer and cardiac genes. In addition, since the beating frequency of EBs generated under reserpine was significantly reduced, one might assume deteriorated functional expression of ion channels or subsequent pathways during cardiac development [31]. Due to the low chemical stability of catecholamines their absolute concentrations were not accessible in the present work. Instead, we used MEA measurements to functionally study the reserpine-induced depletion of catecholamines by recording the reserpine-induced negative chronotropy (Fig. 6, table 2).We found that the adrenergic receptor agonists NE, EPI and ISO acutely rescued the reserpine-induced negative chronotropic effect. We therefore conclude that the inhibiting effect of reserpine on cardiac development has been caused by catecholamine depletion, emptying of catecholamine stores and subsequent decreasing stimulation of ARs. In line with that assumption we found that α- and β-antagonists mimicked the reserpine effect on cardiac development.

Several studies were performed investigating effects of murine genetic knock-outs of enzymes involved in catecholamine synthesis showing severe developmental defects. In 1995, Thomas and colleagues reported that in DBH knock-out mice most of the homozygous embryos died *in utero*, most probably due to cardiovascular failure [10]. Being unable to synthesize NE and EPI, these homozygous mice might resemble the situation in reserpine-treated EBs. Since embryos died even before formation of adrenal glands and maturation of the sympathetic nervous system, Thomas and colleagues suggested different primary sources of catecholamine biosynthesis between embryos and adult mice [10]. Supporting the idea of a crucial role of catecholamines in embryonic development, two groups established mouse models deficient in intact TH and hence depleted of DA, NE and EPI. [9], [11]. While Kobayashi et al. could not identify any “gross deformity” in any of the organs but reduced cardiac beating rate in homozygous neonates, Zhou et al. suggested cardiovascular failure as the cause of embryonic fatality [9], [11]. In line with our observations both groups suggested a paracrine action of catecholamines at a developmental stage before neuronal innervation is established. Deleting PNMT, the enzyme catalyzing the biosynthetic step from NE to EPI, however, did not lead to embryonic lethality in contrast to the effects of TH and DBH deletions [6]. This finding supports the assumption that NE is the most critical catecholamine for cardiac development. Thus, our observation of strongly inhibited cardiac differentiation upon reserpine-induced catecholamine depletion appears plausible. While our *in vitro* ES cell culture system allows defined experimental conditions, the situation in terms of cell/cell interplay, hormonal regulation, and maternal/fetal interaction is far more complex *in vivo* [32]. It seems as if catecholamines had a more pronounced effect *in vitro*. Placental transfer of catecholamines from the heterozygous mothers to homozygous embryos might rescue at least some embryos from prenatal death or may uphold, at least in part, the cardiac differentiation program [9]–[11]. The importance of placental transfer was nicely shown by Thomas et al. in 1995. They reported that 12% of their DBH^−/−^ embryos from heterozygous mothers survived to birth, whereas, all DBH^−/−^ embryos from homozygous mothers died before E13.5 [10]. The same study also nicely showed that heterozygous embryos (DBH^+/−^) from homozygous mothers synthesized NE almost at wild-type levels. Whether maternal or embryonic catecholamines are more important for development remains unclear. If maternal catecholamine levels would *per se* supersede the levels released by the embryo, embryonic cardiac development would be strongly dependent on the mother’s catecholamine levels (e.g. stress exposure, etc.). We, therefore believe, that a local regulatory system might control catecholamine concentrations within the blastocyst and/or the embryo, at least during the earliest steps of cardiac development. In line with this assumption an earlier study demonstrated that excessive exposure to catecholamines or β-adrenergic agonists can also induce cardiac malformation but was demonstrated exclusively at early stages (E4 to E5) of chick development [33]. Accordingly, since in murine gene knock-out models of catecholamine biosynthesis embryonic mortality appeared already between E9.5 and E13.5 [9]–[11], a “critical period” was suggested in which embryos are “hypersensitive” to catecholamine exposure [30]. In summary, since depletion as well as over-application of catecholamines alters cardiac development *in vivo*, a fine interplay of different catecholamines and specific and well controlled catecholamine levels are obviously important for intact cardiac formation and functional development. We were able to show that reserpine not only applied continuously until day 14 but also only until day 6 is sufficient to seriously inhibit cardiac development. This strongly suggests that in our *in vitro* ES cell system, the earliest developmental steps resemble the most critical period for catecholamine-modulated cardiac development [9]–[11]. Furthermore it is well known that *in vivo* and *in vitro* (ES cell) cardiac development is largely analogous (reviewed in [34]).

Catecholamines are known to serve as important regulators of the heart rate even prior to nerval control [1], [2]. Especially ICA cells that have previously been identified in heart tissue of several species [5]–[7] are believed to locally synthesize catecholamines potentially compensating for the rudimentary neuronal adrenergic innervation during earliest steps of development [8]. By immunohistochemical staining with anti-DBH antibodies we found some catecholaminergic DBH expressing cells with slightly elevated numbers in reserpine-treated EBs. As described for ICA cells [7], these cells were small and round but not resembling a neuronal phenotype. Though, looking like ICA cells, these cells were not surrounded by cardiac cells in the reserpine-treated group. So far, mechanisms controlling ICA cell growth and proliferation are unclear. We hypothesize that in the presence of reserpine ICA-like cells are compensatory increased, e.g. by feed-back mechanisms, but still catecholamine-depleted. As consequence the catecholamine effect on cardiac differentiation in reserpine-treated EBs was still diminished despite increase of ICA-like cells..In addition our results show premature and increased expression of catecholamine synthesis enzymes in reserpine-treated cells. As commonly seen in anabolic biosynthetic pathways, low levels of NE and EPI activate compensatory expression of the preceding synthesis enzymes (e.g. *Th*, *Ddc* or *Dbh*). However further experiments are needed to verify this theory.

Adrenergic receptors belong to the G protein-coupled receptor superfamily (GPCRs) [35]. Upon catecholamine binding to their specific receptor subtypes, a variety of second messengers trigger a complex network regulating the activity of proteins involved in almost all areas of cellular function. The complexity of the adrenergic system is not understood completely. Functions can roughly be divided into short-term (e.g. ion channel activity, receptor sensitivity, general metabolism or neurotransmitter synthesis and release) and long-term modulating effects (e.g. synthesis of channels, receptors and intracellular messengers, synaptogenesis and generally gene expression) [36], [37]. We used MEAs to confirm that reserpine-induced depletion of the catecholamine stores (thereby reducing beating frequency of spontaneously beating clusters) is recovered by adrenoreceptor agonist application. We therefore conclude that reserpine-induced cardiac development inhibition is most likely dependent on catecholamine depletion and reduced adrenoreceptor signaling. Several lines of evidence lead to the conclusion that the β_1_- and the α_2_-adrenoreceptor systems are the most likely candidates, because *in vivo* they were shown to negatively impact embryonic development when being deficient [38]–[40]. In line with these findings, results of our experiments applying phentolamine and propranolol to antagonize α- and β-adrenergic receptor signaling showed negative effects on cardiomyogenesis, closely resembling the negative effects of reserpine. These results strongly suggest catecholamine-induced α- and β-adrenoceptor-mediated signaling to be crucial for cardiac development in ES cells which could at least partially be extrapolated to the *in vivo* situation. Our sq-RT-PCR results identified α_1D_, α_2C_ and the β-ARs as possible candidates promoting the catecholamines’ effects on cardiomyogenesis, because they are expressed prematurely in reserpine treated EBs. Most striking however is the rather unexpected strong expression of β_3_ in reserpine-treated EBs. Since except for α_2A_ and β_3_, all ARs are absent at day 10 after reserpine treatment a wide-spread role of catecholamines on the differentiation of a variety of cell types (all those expressing ARs) seems to be likely.

Beside effects of reserpine on cardiac development, we further provide evidence for alterations in the expression of non-cardiac genes. Amongst these alterations in gene expression, up-regulation of ectodermal genes can be directly correlated with neuron-like cells detected in the cell culture. Even in conditions optimized for cardiac differentiation, ES cells treated with reserpine obviously develop into EBs containing remarkable numbers of neurons and cells also appear more mature based on their morphology with typical axonal and dendritic outgrowths. Yet, to our knowledge, evidence of an effect of catecholamines during neuronal development is lacking. So far, *in vivo* studies with heterozygous TH mutant mice (TH^+/−^) with moderate 73–80% of wild-type NE levels revealed impairments in different learning and memory tasks but no morphological changes in brains [41]. However, a possible reason for the positive effect of reserpine on neuronal development could be based on our results showing that reserpine affects expression of early developmental genes, like *Rex1* and *Fgf5,* suggesting a very early effect of catecholamines on cell fate decision. Endoderm and mesoderm are derived from a transient progenitor cell population known as the mesendoderm [42]. In parallel the ectodermal lineage arises, which differentiates into neuronal cells (amongst other cell types) during further development. Thus, the reserpine-induced mesodermal inhibition could indirectly promote neuroectodermal differentiation. Moreover, our results show that *Fgf5* is kept up-regulated at day 6 after the start of expression when cells are treated with reserpine. *Fgf5* was not only described as a marker for the embryonic gastrulation, but its expression has also been suggested to be somehow linked to ectodermal germ layer cells and to also promote commitment of undifferentiated cells to neuronal lineages [26], [43]. Taken together, our findings suggest that reserpine might influence neuronal cell fate decisions directly. Moreover, qPCR results presented here indicate, that between day 2 and day 4 *Rex1* is strongly down-regulated in reserpine-treated cells, whereas *Oct4* expression is similar to controls. In a recent publication evidence was shown that *Rex1^−/^Oct3/4^+^* cells undergo differentiation into neuroectoderm more efficiently than *Rex^+^/Oct3/4^+^* cells [44]. Therefore, the *Rex1/Oct4* ratio found in reserpine-treated cells resembles a *Rex1^−/^Oct3/4^+^* situation, thus, potentially promoting more efficient differentiation into neuroectoderm, suggesting that increased neuronal cell numbers in reserpine-treated EBs may be caused by early cell fate decision changes.

In the present study we describe that reserpine inhibits cardiac differentiation of murine ES cells without affecting cell proliferation. Comparing our ES cell system to earlier published catecholamine synthesis deficient *in vivo* models reveals not only parallels but also distinct differences. Catecholamines do promote cardiac differentiation in EBs, underlining the comparability of *in vivo* models with ES cell models. In addition, we could show that they influence a broad range of cellular mechanisms. But intriguingly, we found that reserpine application increased the numbers of neuronal cells in EBs. A role of catecholamines on ectodermal and neuronal differentiation has not been shown so far. We assume that our results will help to gain deeper insight into the role of catecholamines during ES cell differentiation providing potentially new targets for directed stem cell differentiation protocols. Our data suggest that high dose α- and β-blocker therapy during early pregnancy needs clear indications [45].

## Supporting Information

Figure S1 **Quantification of eGFP positive CMs in EBs.** Representative flow cytometry derived dot plots of (A) untreated and day 0–14 DMSO- and reserpine-treated EBs at day 10 and day 14 (B) EBs treated with DMSO or reserpine from day 0–6 (until d6) at day 10 and day 14.
(TIF)

Figure S2 **EB size measurement at day 2 and day 4.** Microscopy pictures of control, DMSO and reserpine-treated EBs at day 2 and 4 as used for cross-section area analysis for Fig. 2C. Scale bars: 50 µm.
(TIF)
