# Supplementary figures and images for: Evidence for a Critical Role of Catecholamines for Cardiomyocyte Lineage Commitment in Murine Embryonic Stem Cells
Source: PLoS One. 2013 Aug 2;8(8):e70913. doi: 10.1371/journal.pone.0070913 (PMC3732289; doi:10.1371/journal.pone.0070913)

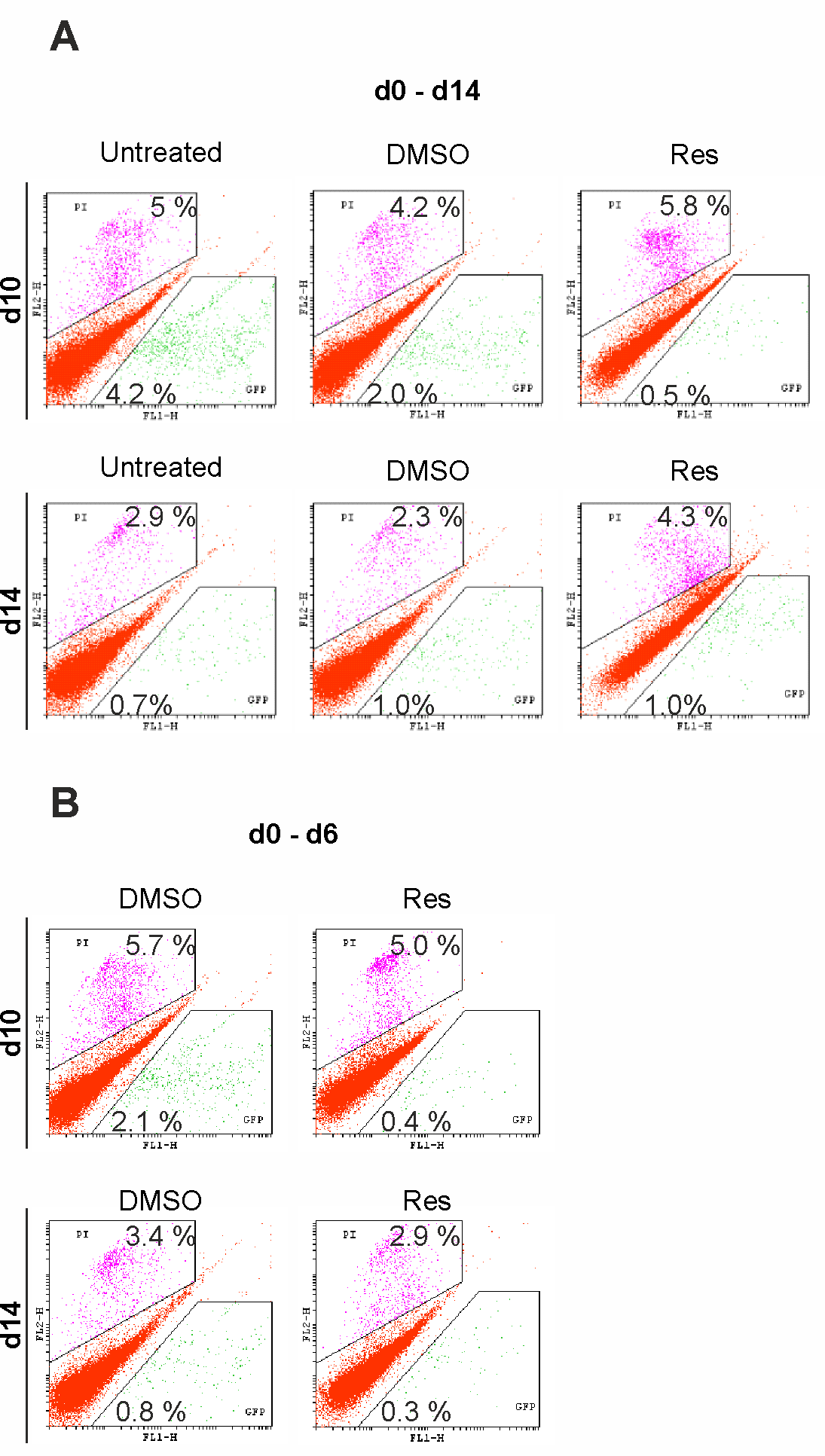

Supplement: Figure S1 — Quantification of eGFP positive CMs in EBs. Representative flow cytometry derived dot plots of (A) untreated and day 0–14 DMSO- and reserpine-treated EBs at day 10 and day 14 (B) EBs treated with DMSO or reserpine from day 0–6 (until d6) at day 10 and day 14. (TIF) [file pone.0070913.s001.tif]

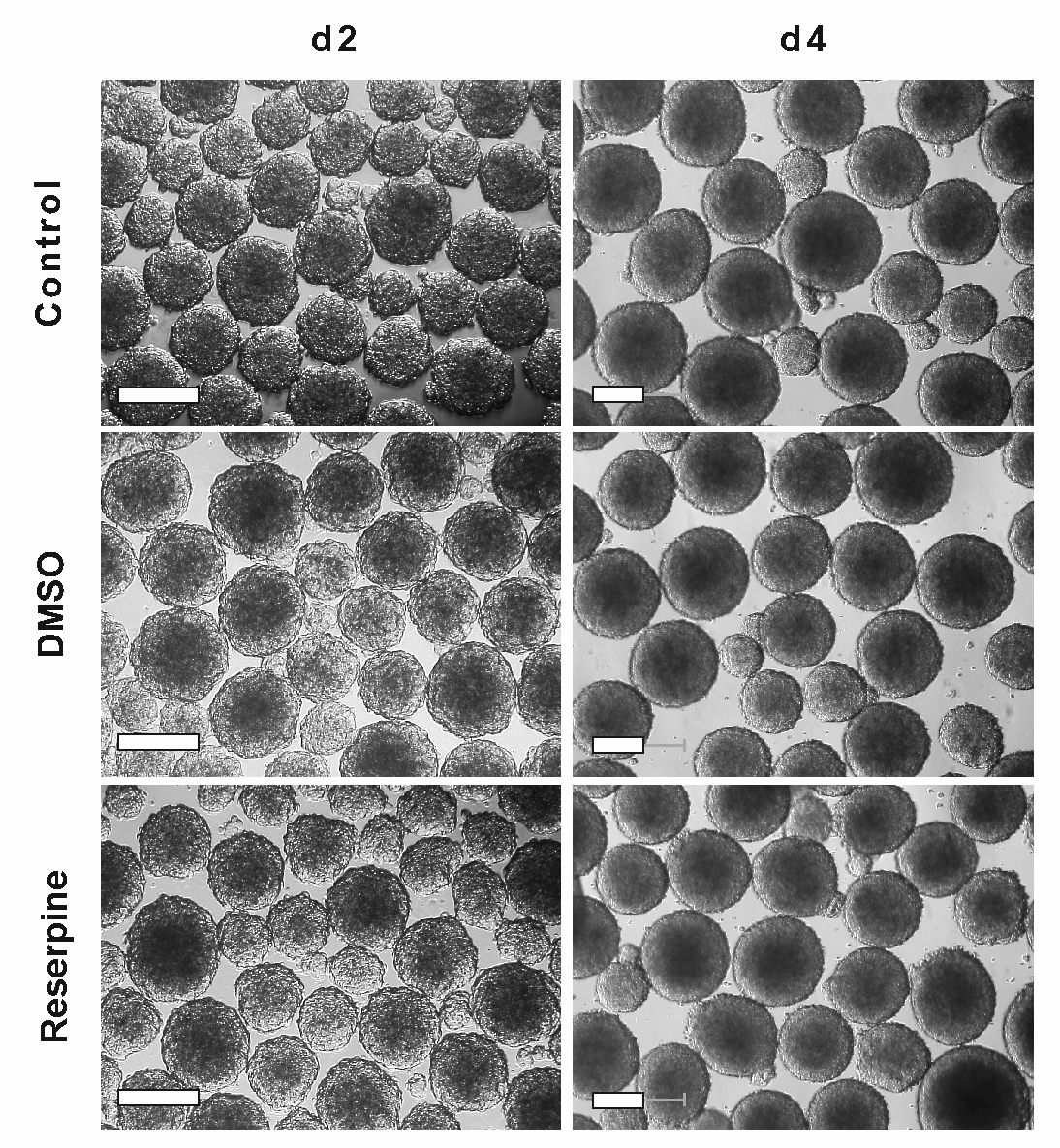

Supplement: Figure S2 — EB size measurement at day 2 and day 4. Microscopy pictures of control, DMSO and reserpine-treated EBs at day 2 and 4 as used for cross-section area analysis for Fig. 2C. Scale bars: 50 µm. (TIF) [file pone.0070913.s002.tif]
